# Genome-Scale Screening of Drug-Target Associations Relevant to K_i_ Using a Chemogenomics Approach

**DOI:** 10.1371/journal.pone.0057680

**Published:** 2013-04-05

**Authors:** Dong-Sheng Cao, Yi-Zeng Liang, Zhe Deng, Qian-Nan Hu, Min He, Qing-Song Xu, Guang-Hua Zhou, Liu-Xia Zhang, Zi-xin Deng, Shao Liu

**Affiliations:** 1 Research Center of Modernization of Traditional Chinese Medicines, Central South University, Changsha, P. R. China; 2 Key Laboratory of Combinatorial Biosynthesis and Drug Discovery (Wuhan University), Ministry of Education, and Wuhan University School of Pharmaceutical Sciences, Wuhan, P. R. China; 3 The 163rd Hospital of The Chinese People's Liberation Army, Changsha, P. R. China; 4 School of Mathematics and Statistics, Central South University, Changsha, P. R. China; 5 Xiangya Hospital, Central South University, Changsha, P. R. China; University of Rome, Italy

## Abstract

The identification of interactions between drugs and target proteins plays a key role in genomic drug discovery. In the present study, the quantitative binding affinities of drug-target pairs are differentiated as a measurement to define whether a drug interacts with a protein or not, and then a chemogenomics framework using an unbiased set of general integrated features and random forest (RF) is employed to construct a predictive model which can accurately classify drug-target pairs. The predictability of the model is further investigated and validated by several independent validation sets. The built model is used to predict drug-target associations, some of which were confirmed by comparing experimental data from public biological resources. A drug-target interaction network with high confidence drug-target pairs was also reconstructed. This network provides further insight for the action of drugs and targets. Finally, a web-based server called PreDPI-K_i_ was developed to predict drug-target interactions for drug discovery. In addition to providing a high-confidence list of drug-target associations for subsequent experimental investigation guidance, these results also contribute to the understanding of drug-target interactions. We can also see that quantitative information of drug-target associations could greatly promote the development of more accurate models. The PreDPI-K_i_ server is freely available via: http://sdd.whu.edu.cn/dpiki.

## Introduction

The identification of drug-target interaction networks is an area of intense research in drug discovery [1], [2], [3]. The emergence of molecular medicine and the completion of the human genome project provide more opportunity to discover new drug targets. Much effort has been made in the past few years to achieve this goal. There are thousands of FDA-approved drugs on the market and potential drugs in the later phases of clinical trials. The identification of drug-target interactions helps researchers to find new targets for an old drug as well as new drug candidates for a drug target [4]. Finding potential applications in other therapeutic categories of those FDA-approved drugs by predicting their targets, known as drug repositioning, is supported by the core observation that a single drug often interacts with multiple targets [5]. It offers an appealing strategy, and can be regarded as a very efficient and time-saving method in drug discovery [6], [7], [8]. The identification of potential targets for a drug provides insights into its potential toxicity and/or its new application to the therapy of other diseases. Additionally, predicting drug-target interactions could help decipher the underlying biological mechanisms from the network perspective [9], [10], [11]. More importantly, the determination of drug-target interactions remains very challenging and time-consuming at the experimental level. It is almost impossible to carry out all experiments detecting the toxicity of a drug candidate by checking the interactions between this candidate and related proteins.

Currently, two computational approaches are generally used for studying the drug-target relations: ligand-based virtual screening and docking. The ligand-based approach is to predict the drugs interacting with a given protein based on the chemical structure similarity in a classic SAR framework. Keiser et al. proposed a method to predict protein targets based on the chemical similarity of their ligands [12]. Likewise, Campillos et al. used side effect similarity to relate drugs to novel targets [13]. These two kinds of approaches, however, do not take advantage of the information in the protein domain. Docking is a powerful molecular modeling approach that predicts the preferred orientation of a drug molecule to a protein by dynamic simulation, and a series of ranked drug-target relations can be generated by the size of energy scores [14], [15], [16], [17]. However, a major limitation is that docking approaches need 3D structures of proteins. Moreover, the problem is especially serious for membrane proteins, e.g., very few GPCRs have been crystallized. Recently, Several statistical methods have been developed to predict compound – protein interactions [18], [19], [20], [21], [22]. An example was the pairwise kernel that measures the similarity between drug-target pairs [23], [24]. However, the drawback of the pairwise kernel is that there will be a large number of samples to be classified (i.e., number of drugs multiplies number of targets) which poses remarkable computational complexity. Another problem is that the negative drug-target pairs are selected randomly without experimental confirmation. More recently, Bleakley et al. proposed a bipartite local model by transforming edge-prediction problems into binary classification problems [25]. Laarhoven et al. developed a Gaussian interaction profile kernel for predicting drug-target interactions [26].

It is worth noting that, among these prediction methods, the quantitative information of drug-target pairs was not taken into account. It seems preferable that the classifier predicts not only whether one drug-protein pair has an interaction or not, but also whether this pair has a stronger interaction or not. A considerable portion of drug discovery focuses on lead finding and optimization by evaluating its affinity to the primary target [27]. In fact, pharmacologists are more interested in those drug-target associations with strong binding affinities, which are a good starting point for further experimental research [28]. K_i_ is the inhibition constant for a drug; the concentration of competing ligand in a competition assay which would occupy 50% of the receptors if no ligand is present. K_i_ can quantitatively describe the degree to which the drug binds to the target protein. Distinguishing tight binding from moderate binding (nM vs μM level inhibitors) is an urgent task. If this was overcome, one could identify candidate compounds over a handful of leads and significantly reduce false positives. Also, more accurate modeling by quantitative biochemical data on targets takes us one step closer to predict selectivity, toxicity and druggability [29]. Based on recent studies in systems biology, it is possible to see that quantitative data will inform models of drug action and uncover new pharmacological hypotheses. In this study, we try to make full use of quantitative drug-target interactions to construct a predictive model and to avoid some problems such as unknown drug-target interactions, -being assumed as non-interaction.

In this article, we present a discriminative computational framework to identify drug-target associations in human species by developing a chemogenomics approach using integrated molecular features and K_i_ [30]. We aim at integrating chemoinformatics (e.g., drugs) and bioinformatics (e.g., targets) into an interaction informatics platform for genomic drug discovery. We used a random forest (RF) model to differentiate drug-target interactions from non-interactions or tight binding from moderate binding. RF has been successfully applied in many biological contexts: cancer tissue classification [31], [32], [33], protein domain classification [34], nucleosome positioning, etc. In our case, because of potentially diverse mechanisms between drugs and targets, we use a complete set of drug-target interaction features to predict new drug-target associations. To demonstrate the reliability of our methodology, we investigate the discriminative models using only drug features, target features and integrated features, respectively. In addition to five-fold cross validation, we evaluate our method by predicting drug-target pairs from external validation sets collected from public resources.

We further apply our RF approach to predict putative drug-target interactions. Many of novel drug-target pairs obtained high prediction probability and some have been validated from public biological resources. Network reconstruction with high confidence drug-target pairs provides an impressive picture about drug-target associations, helping in further understanding drug and target actions [35], [36]. The successful identification of tight binding drug-target associations provides powerful independent evidence for the validity of our approach. The interactions can provide complementary and supporting evidence to experimental studies. Finally, a web-based drug-target prediction server was established to facilitate the use of scientific researchers.

## Results

### Drug-target interactions can be accurately predicted from integrated features

Our primary concern is to construct a predictive model that can accurately differentiate drug-target interactions with strong binding affinity from those with weak binding affinity, and to investigate the degree to which we can predict drug-target interactions in human species using integrated features. We mainly focus on the drug-target pairs available on two popular databases: the Binding database and the PDSP K_i_ database. As a starting point of investigation, cross-linking associations in human species were extracted. We initially chose a commonly used K_i_ threshold to tune the positive set (drug-target pairs with K_i_ value < the given threshold) and the negative set (drug-target pairs with K_i_ value ≥ the given threshold). In practice, 10 μM K_i_ value is usually used as a critical value to differentiate whether one drug-target pair interacts or not [3]. Thus, the entire data set was firstly divided into 8745 positive samples and 4334 negative samples. We initially used this K_i_ value to assess the predictive capability of our constructed model.

**Figure 1:**
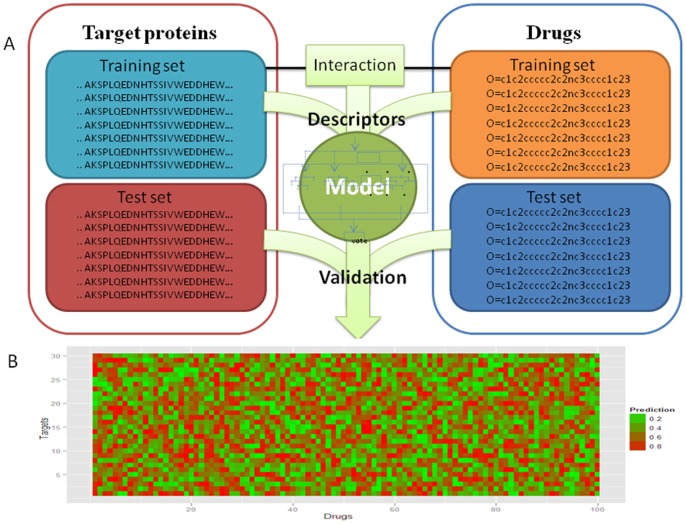
Outline of our methodology. (A) Interaction features are calculated by combing the fingerprint descriptors from drugs and the CTD and amino acid composition descriptors from protein sequences. These feature vectors are used to find the optimal RF parameters which most accurately separate the positive and negative training sets. The independent validation sets are used for further validation for the RF model. (B) Once the RF model is constructed, we can predict new unknown drug-target associations or screen all cross-linking associations.

To represent drug-target interactions, we used a chemogenomics framework. In brief, an interaction could be efficiently represented by simultaneously considering drug descriptors and protein descriptors. The basic approach is outlined in **Figure 1A**. In our approach, drug molecules were represented by chemical hashed fingerprints of a 1024 bits length [37]. Target proteins were represented using composition, transition and distribution (CTD) descriptors and amino acid composition descriptors (167 descriptors). Thus, each interaction sample (positive or negative) was finally characterized as a 1024+167 = 1191 dimensional vector by concatenating drug descriptors and protein descriptors. Each of these factors could be considered as a separate coordinate spanning a multidimensional space, and in this sense a drug-target interaction is an event in this type of multidimensional space. We used the full set of 1191 descriptors as our model input. Because many drug-target interactions have yet to be determined, we prefered the application to an unbiased, general, and complete set of molecular features. The result of scanning unknown drug-target pairs is shown in **Figure 1B**, and the result of identifying novel and experimentally confirmed associations is discussed in detail below.

**Figure 2:**
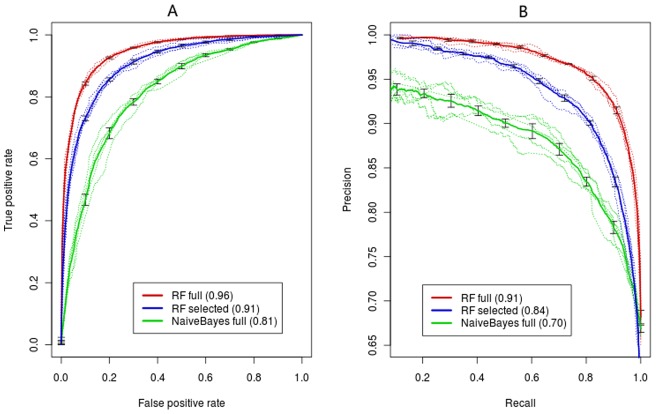
ROCs and precision-recall curves for Naïve Bayes (green) and random forest (red) with full and selected features. (A) ROCs (B) precision-recall curves.

To evaluate classification performance, we first used a five-fold cross validation method. Initially, the whole data set to be classified was randomly partitioned into five subsets. One subset was then reserved as a validation data set, and the classifier was trained in the remaining four subsets. The constructed classifier was then used to predict the reserved validation data set to assess its accuracy. The process was repeated five times so that every drug-target association was classified. Because there is a trade-off between sensitivity and specificity, we measure the quality of the classifier by calculating the area under the ROC curve (auROC), as shown for K_i_ = 10 μM threshold in **Figure 2**. An ROC curve shows the false-positive rate along the *x*-axis and the true-positive rate along the *y*-axis, as the classification threshold varies for declaring a prediction to be a real site [38]. A model with no predictive ability would yield the diagonal line. We ultimately averaged five validation set auROCs to obtain a summary statistic of classification performance [39].

We employed the random forest (RF) algorithm to construct our predictive model because of its excellent reputation amongst the bioinformatics communities [40], [41], [42]. In RF, two parameters, the number of randomly selected variables *mtry* and the number of trees grown *ntree*, needed to be further optimized. To achieve the better performance, we screened *mtry* values ranging from 5 to 100 with a step of 5. We also selected an appropriate number of trees to be grown to achieve a low error rate of convergence. Ensemble of 600 trees (*ntree*  = 600) was finally used to construct RF (**Figure S1**). All optimal models were determined using five-fold cross validation before proceeding to prospective validation of the model. In addition, we tested and compared a commonly used alternative approach, the Naïve Bayes classifier, which learns these parameters for each feature independently (the RF classifier learns the interaction of features at the same time). Despite this assumption of independence, the Naïve Bayes classifier has performed very well on a broad range of machine learning applications.

To test the sensitivities of various K_i_ thresholds with our RF model, we repeated these cross validation experiments on different positive/negative sets divided by different K_i_ thresholds ranging from 10 to 90 with a step of 5. Although the ROC curve is a standard metric, the precision-recall (P-R) curve is a more reliable measure of performance than the ROC curve. Precision is the ratio of true positives to predicted positives, and recall is identical to the true positive rate in the ROC curve. The P-R curve can be quantified by the area under the P-R curve (auPRC), or average precision.

**Figure 3:**
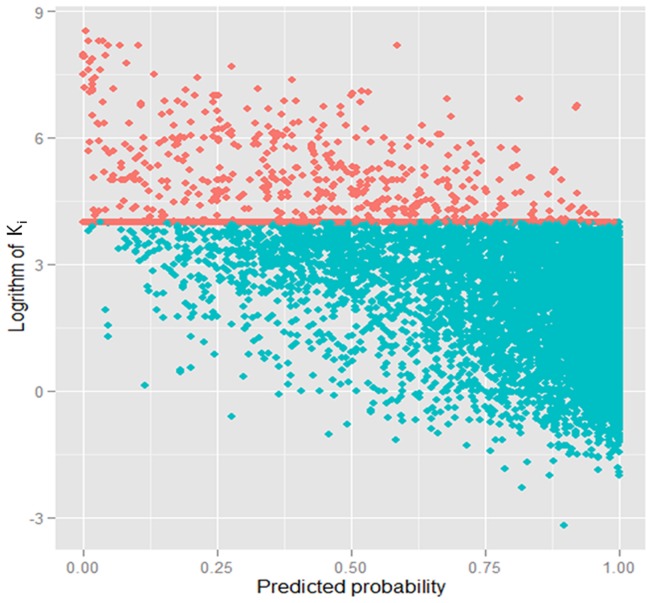
The plot of K_i_ versus prediction probability on 5-fold cross validation. non-interaction: red and interaction: green. Linear relationship between K_i_ and prediction probability could be observed with correlation coefficient of 0.65.

Our main result is that using this K_i_ threshold (10 μM), the RF can successfully distinguish the drug-target interactions with auROC  = 0.96 and auPRC  = 0.91, and prediction accuracy of 88.74% can be obtained (see **Table 1**). The ROC curve reveals a sensitivity of 90% at a false positive rate of 18%. This is significantly better than the false positive rate of 90% from random predictions at this sensitivity (*p* -value <10e-78). The Naïve Bayes classifier is significantly less accurate in distinguishing the drug-target interactions (auROC  = 0.81 and auPRC  = 0.70), indicating that the assumption of conditional independence among interaction features impairs its performance. **Figure 2** shows the summaries of comparison between auROCs and auPRCs of RF and Naïve Bayes. Observation of error bars for two curves found that RF is more robust than Naïve Bayes. In **Figure 3**, we plot the figure of K_i_ versus prediction probability on five-fold cross validation. Clearly, a significant trend can be found that K_i_ values increase as prediction probabilities decrease. The linear relationship with correlation coefficient of 0.65 can be found (*p*-value <2.2e-16). This indicates that the drug-target pairs with tight binding (low K_i_ values) have high prediction probabilities, and vice versa. Further analysis found that the drug-target pairs predicted wrongly are located in the range from 2 to 6 (the logarithm of K_i_). For example, for positive samples, 89% of positive samples predicted wrongly are located in the range from 2 to 4, again implying that the drug-target pairs at the classification margin are more difficult to distinguish.

**Table 1:** Prediction results of five-fold cross validation using different models.

	TP	FN	TN	FP	Sen	Spe	Acc	auROC	auPRC
RF	8003	742	3603	731	91.52	83.14	88.74	95.84	91.04
Naïve Bayes	7212	1533	3134	1200	82.47	72.32	79.10	81.47	70.32
RF-drug	7648	1097	3447	887	87.46	79.54	84.83	88.12	79.28
RF-target	7838	907	2155	2179	89.63	49.72	76.40	73.35	63.57
BGL	5661	3084	4274	60	64.74	98.62	75.96	90.42	82.27

TP: true positives; FN: false negatives; TN: true negatives; FP: false positives; Sen: sensitivity; Spe: specificity; Acc: accuracy.

**Figure 4:**
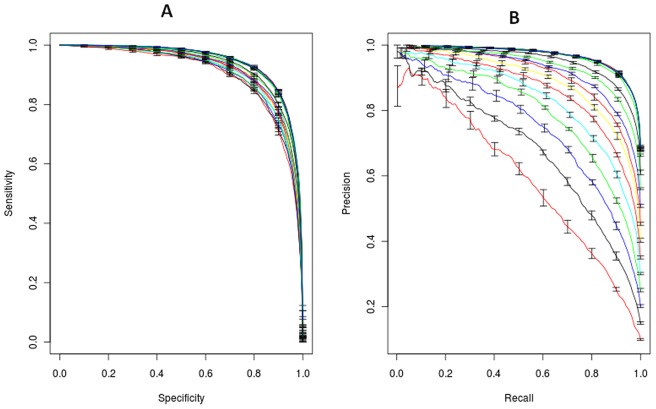
ROCs and precision-recall curves with different K_i_ thresholds using RF. (A) ROCs (B) precision-recall curves. The auPRCs drop with the decreasing of K_i_ thresholds. However, the varying trend of auROCs is consistent with that of auPRCs.

For classification of drug-target pairs tuned by different K_i_ values, auROC is almost unaffected by the K_i_ threshold (**Figure 4A**), but auPRC drops (**Figure 4B**) as K_i_ threshold decreases. However, the trends of auROC and auPRC are consistent. The densities of prediction probability using varying K_i_ thresholds are plotted in **Figure S2**. The trend in this plot is consistent with the one in the P-R curve. It can be seen that the optimal discriminative value increases from about 0.1 to 0.6 as the K_i_ threshold increases. Furthermore, the choice of large K_i_ threshold significantly increases the number of positive samples with high prediction probability. Taken together, these results indicate that the degree to which drug-target pairs are successfully predicted is dominated by their binding affinities. The model using quantitative information on targets can efficiently differentiate interactions from non-interactions, even strong binding from weak binding.

### The features from drugs and target proteins collectively contribute to the discrimination of drug-target interactions

Next, we investigate which subsets of features allowed RF to successfully discriminate drug-target interactions. The RF itself has a built-in feature evaluation program that allows user to rank features. We conduct the RF classification again, using only the subset of integrated features with large importance. The RF using the top 300 features achieves auROC of 0.91. This indicates that the features with larger importance predict drug-target interactions with similar accuracy, although the auROC slightly decreases compared to the result with the full set of features (**Figure 2A**). However, we failed to find single feature that greatly contributes to our discrimination. By combining many features, the full RF and the RF with the top 300 features achieve greater accuracy than single feature. The RFs outperform the Naïve Bayes that assumes features independence, which indicates that these features contribute cooperatively. We also investigate how the omission of protein characteristics can affect the performance. Information of a protein property is omitted at every turn (**Table S2**). Each omission affects performance only mildly, suggesting that none is very critical to our performance, but each improves it slightly (**Figure S3**). This may imply complicated interactions in drug-target pairs and reflects the difficulty of interpreting specific molecular feature to a certain degree.

A striking feature of our approach is that integration of information from drugs and targets are quite helpful for representing the drug-target associations. We assume that drug-target interactions can be determined by structural features from drugs and targets, which comprise of a pharmacological space. Chemogenomics research aims to relate the chemical space with the genomics space in order to identify potentially useful associations in the pharmaceutical space. To demonstrate the reliability of our assumption, we re-establish our RF model using only the structural content from single space (i.e., chemical space or genomics space), that is, two RF models are constructed using 1024 drug features and 167 protein features, respectively. As can be seen from **Figure S4**, the RFs with 1024 drug features and 167 protein features obtain relatively inferior prediction (auROC: 0.88 vs 0.73), respectively (see **Table 1**). The comparison between RFs with separate spaces and RF with integrated features indicates that the structural contents from drugs and targets contribute to the discrimination of drug-target associations cooperatively.

### Random forest model validation using external validation sets

To further demonstrate the prediction ability of the models, they should also be validated by predicting the interactions of other drug-target associations not used in the training set, but whose interactions have been experimentally determined (i.e., independent validation set). Herein, six independent validation sets are employed (see Methods section). The results that applied our RF to these validation sets are listed in **Table 2**. For validation set 1, 1829 drug-target pairs are successfully predicted from 2041 associations and prediction accuracy of 89.61% is obtained. For validation set 2, we successfully predict 4155 drug-target pairs, and prediction accuracy of 81.04% was achieved. Considering more number of targets than those from the reference set, our prediction for this validation set seems satisfactory. Validation set 3 is a larger validation set from the ChEMBL database compared to the above two validation sets. Our prediction reveals that 23674 out of 30102 drug-target associations are correctly predicted and prediction accuracy of 78.64% is obtained. Validation sets 4 and 5 are used to demonstrate the ability of our model to discriminate the protein-ligand complexes from decoy complexes. The results effectively illustrate predictability of our approach (94.61% for protein-ligand complexes versus 82.76% for decoy complexes). For validation set 6, these associations are identified by some non-structural similarity inference way. We aim at using these to validate the assumption of our model (i.e., structural similarity principle). Finally, we successfully identify 31 out of 43 interaction pairs. Such a result indicates that our approach has the ability to identify those associations found by indirect inference to a certain degree. Although predicting some associations correctly, we also find that these predictive probabilities are relatively low. Our model may be insufficient to identify such associations because it is based on only structural similarity principle. The predictions from these validation sets, together with those from cross validation, collectively demonstrated that our approach is able to accurately predict drug-target associations.

**Table 2:** Prediction results for independent validation sets by RFs.

	Total number	Predicted correctly	Accuracy
Validation Set 1	2041	1829	89.61%
Validation Set 2	5127	4155	81.04%
Validation Set 3	30102	23674	78.64%
Validation Set 4	334	316	94.61%
Validation Set 5	1560	1291	82.76%
Validation Set 6	43	31	72.09%

### Genome-wide RF predictions identify novel drug-target associations

**Figure 5:**
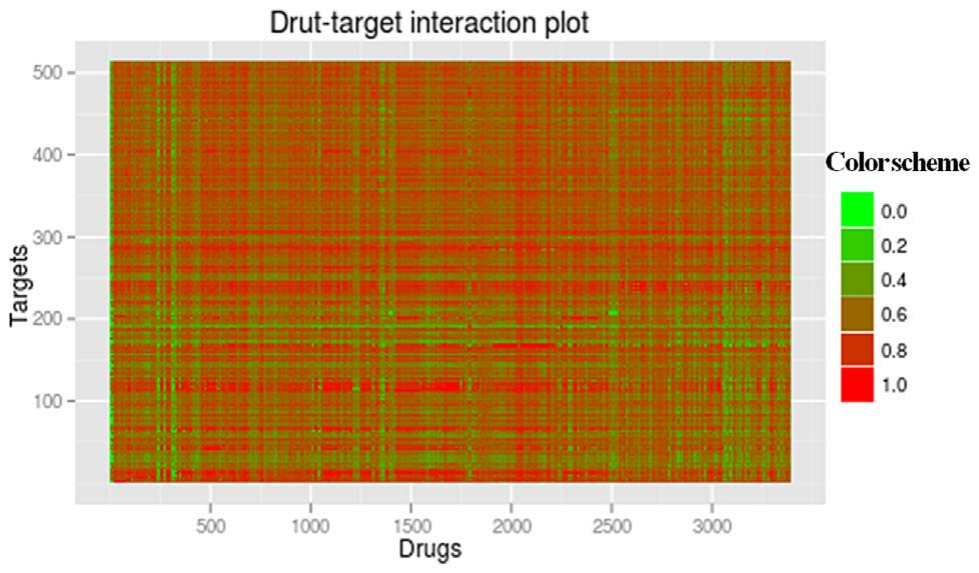
The predictive probability plot of screening all cross-linking drug-target pairs. The size of predictive probability gradually varies from green to red.

To predict additional drug-target pairs that are not included in our training set, we scan the entire drug-target associations systematically with our RF. Thus, a 514×3393 prediction matrix is finally obtained to record prediction probabilities. We found the significant enrichment of drug-target associations according to our RF prediction probability (**Figure 5**). By using alternative thresholds, our approach may be tuned to predict a subset of drug-target pairs with high confidence at the cost of a false discovery rate (the expected fraction of predicted positives which are false positives, FDR = FP/(FP+TP)). We can estimate FDR from the P-R curves in **Figure 2B**. For example, 83% of the interactions are detected at a FDR of 5% (*p*-value <10e-93). To trade off precision and recall, we choose a cutoff which corresponds to 45% recall, which at 10 μM K_i_ threshold is RF prediction probability of 0.95. For large K_i_ thresholds, precision is about 99% when recall is 45%, and therefore we estimate our FDR to be about 1%. In other words, at this cutoff (RF >0.95), on the training set, we capture 45% of the drug-target interactions. Although there is a small recall at this FDR, we could guarantee better precision and higher confidence drug-target pairs predicted by our approach. Certainly, we can alter FDRs to obtain different success rates, as listed in **Table 3**.

**Table 3:** Prediction statistics on different false discovery rates.

FDR	Recall	Threshold	Number	Ratio
0.3%	25.60%	0.993	3786	0.22%
0.5%	32.40%	0.990	4343	0.25%
1.0%	45.20%	0.950	11907	0.68%
1.5%	56.50%	0.910	27252	1.56%
2.0%	61.54%	0.880	47160	2.70%
2.5%	66.07%	0.850	77543	4.44%
3.0%	69.93%	0.830	106799	6.12%
3.5%	74.60%	0.790	195722	11.12%
4.0%	77.77%	0.760	293383	16.82%
4.5%	80.21%	0.740	374204	21.46%
5.0%	82.58%	0.710	515375	29.55%

FDR: false discovery rate, Number: Number of drug-target pairs predicted as interactions, Ratio: the ratio between drug target pairs predicted as interactions and all screening pairs on specific FDR.

At a RF threshold of 0.95, we predict 11907 drug-target interactions, and 5987 new drug-target interactions after excluding those appearing in the training set. We expect about 5927 of these associations to be true drug-target interactions. These associations only take up 0.68% of all cross-linking associations. This threshold appears to be a good trade-off for identifying many biologically significant drug-target interactions with an acceptable FDR. The full list of these associations together with their prediction probabilities is included in **Supporting Material J in File S1.** Further analysis reveals that 2191 drug-target pairs obtain prediction probability of 1.0. After excluding 1973 pairs in training set, we obtain 218 new predictions. We are more confident that these drug-target pairs should be correctly predicted. The RF classifier identifies more drug-target associations in the cross-linking set than the training set. This may be due to two factors: (1) These predicted pairs may be false positive associations; (2) They may be true positive associations that are unidentified by binding assays. However, we believe that these associations should be novel interactions at such a small FDR. Thus, when experimental resources are limited and even a few drug-target interactions would be valuable, our method can provide a list of candidate drug-target pairs that is highly enriched for drug-target interactions.

To comprehensively assess the validity of our RF, we manually search in the literature, databases and find some drug-target interactions published as supporting of our predictions [43], [44]. Herein, we only search the first 775 predicted drug-target pairs with prediction probability >0.99. These associations together with the retrieved K_i_ values can be found in **Supporting Material K in File S1.** Clearly, most of these associations have been validated from experiments, demonstrating the predictability of our approach. In summary, our RF model has the ability to predict those drug-target interactions which are still not determined from experiments.

### Network construction of Drug-target associations with high prediction confidence drug-target pairs

We construct a drug-target network using those pairs with high confidence to comprehensively understand the drug and protein action. To guarantee the reliability of our constructed network, we limit FDR at the level of 0.5% (i.e., RF >0.99). Thus, the total set of 4343 drug-target pairs is reliably predicted to be interactions. After excluding 3628 associations in the training set, we additionally predict 775 new associations involving 67 targets and 517 drugs, about 63% of which have been validated from public biological resources (see **Supporting Material K in File S1**). It should be noted that we do not intend to construct a whole network, but to conveniently observe the network action of our predicted drug-target pairs (see also **Figure S5** for total network).

**Figure 6:**
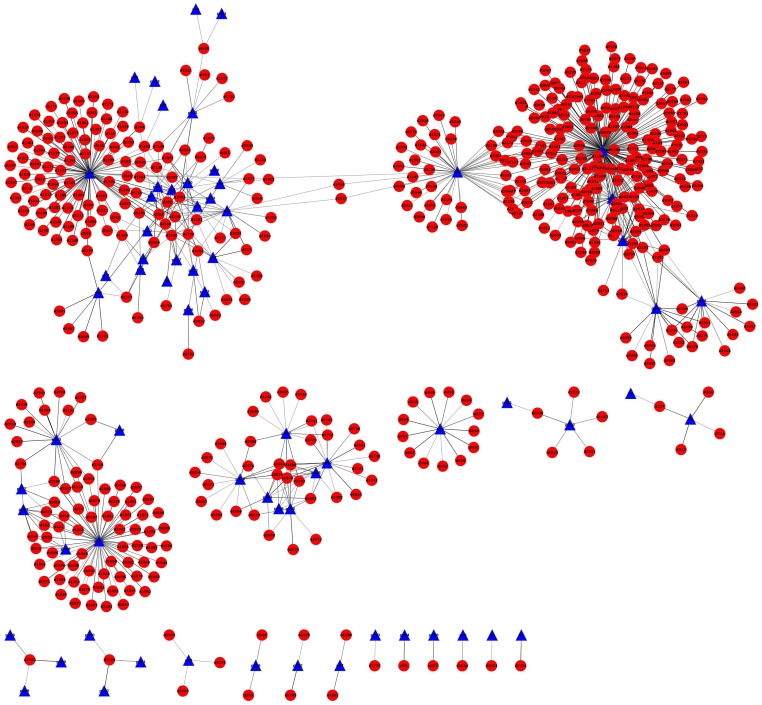
Drug-target interaction network using drug-target pairs with prediction probability above 0.99. Drugs and targets are presented by red circle and blue triangle, respectively. Drug-target interactions are represented by the edges connecting related drugs and targets.

**Figure 6** shows the predicted drug-target network using these 775 interactions. Significant features from the network can be found: (1) It is clear from data mining of binding affinities between drugs and targets that many drugs show clinically relevant polypharmacology (that is, they are ‘dirty drugs’) [45]. There are four large hubs corresponding to different target clusters, and highly connected nodes in the network. They almost take up >95% of all interactions. This indicates high binding affinity for some specific class of targets, such as delta opioid receptors and dopamine receptors. Quite expectedly, closely related members of the gene family will show significant drug promiscuity, and as a result of the generally similar function of these proteins, give rise to complex clinical pharmacology. This could be very well illustrated by biogenic amine receptors. For example, clozapine has a highly complex pharmacological profile, with high affinities for serotonin receptors (5-HT_2A_, 5-HT_2C_, 5-HT_6_ and 5-HT_7_), dopamine receptors (D2, D4), adrenergic receptors (α1- and α2-subtypes) and other biogenic amine receptors [46]. (2) Delta opioid receptor possesses the largest number of connections with drugs, such as opioid analgesics (*e.g.*, nalbuphine, dermorphin, butorphanol, cyclorphan, buprenornhine, diprenorphine, phenazocine, bremazocine), and opioid receptor blockers (e.g., nalmefene, naloxone, clocinnamox, naltrexone, naltrindole, hydromorphone). Most of these drugs have been successfully applied in preclinical or clinical therapy. Among all predictions, we found that delta opioid receptor-drug interactions are very strong since most of their associations have high prediction confidence (prediction probability of 1.0) [47], [48]. Search for K_i_ in PubChem and ChEMBL databases has also demonstrated strong interactions between delta opioin receptor and these drugs (that is, most of drugs are at nM level). Other targets cluster with delta opioid receptor include mu opioid receptor, cannabinoid CB1 receptor, pregnane X receptor, delta opiate receptor and mu opiate receptor. These targets have similar biological function. (3) Serotonin receptors (5-HT_1A_, 5-HT_1B_, 5-HT_1D_, 5-HT_2A–2C_, 5-HT_6_ and 5-HT_7_), alpha adrenergic receptor (e.g., 1A, 2A, 2B and 2C), cholinergic receptors (e.g., M1–M5), histamine receptors (e.g., H1, H3) and dopamine receptors are cross-linked together. Dopamine receptors keep the largest number of connections. Dysfunction of dopaminergic neurotransmission in the CNS has been implicated in a variety of neuropsychiatric disorders, including anxiety disorder, social phobia, Parkinson's disease, schizophrenia, neuroleptic malignant syndrome, attention-deficit hyperactivity disorder, and drug/alcohol dependence. Clearly, most of presently used antipsychotic drugs have a complex pharmacology, with appreciable affinities for a variety of biogenic amine receptors [49]. Recent studies have implicated that histamine receptor H1, the 5-HT_2C_ receptor and α1-adrenoeptors – sites for which many antipsychotic drugs have high affinity – for causing weight gain and associated metabolic side effects [50]. It is found that most of drugs connected to dopamine receptor are drugs used for anxiety disorder (e.g., fluphenazine, sertindole, thioridazine, and trifluoperazine), for Parkinson's disease (e.g., pergolide, lisuride, and apomorphine), for alcohol and drug dependence (e.g., terguride), and antipsychotic drugs (e.g., fluspirilene, loxapine, and clozapine). Some of them as landmark drugs have been routinely applied in practice. (4) The third largest cluster is some receptors related to hormones, such as glucocorticoid receptor, progesterone receptor, androgen receptor, mineral corticoid receptor and so on. Corresponding drugs include CP-409069 (glucocorticoid receptor modulator, antiobesity drugs), mifepristone (progesterone inhibitor), prednisolone, dexamethasone, and their derivatives and so on, which have been approved to treat related diseases. The fourth largest cluster is carbonic anhydrase, which is mainly the binding site for antiglaucoma agents (e.g., benzolamide, dorzolamide), and diuretics (e.g., acetazolamide, furosemide). In summary, the network analysis provides further insights into drug action and target action such as target binding, drug selectivity, polypharmacology and toxicity, although these clinically used drugs have been routinely used.

### Comparison to alternative approaches

We also compare our approach to other alternative modeling approaches. Three commonly used machine learning approaches are employed: support vector machine (SVM), standard back-propagation network (BPN), and *k*-nearest neighbor (*k*-NN). The ROC curves are plotted in **Figure S6** for three modeling approaches. As shown in this plot, one can see that three alternative approaches obtained auROCs of 0.95, 0.89 and 0.84, respectively. The results of BPN and *k*-NN are significantly inferior to those of RF and SVM, and SVM gives similar prediction to RF. However, in view of high computational complexity of SVM and various auxiliary equipments of RF, we prefer RF to construct our predictive model. Additionally, we also compared our approach with the recent bipartite graph learning (BGL) model (**Table 1**). Clearly, better prediction performance can be achieved from the RF model.

### Web-based online prediction server – PreDPI-K_i_

To share our results with pharmacologists and chemists, we finally constructed a web-based prediction server: PreDPI-K_i_. The PreDPI-K_i_ can be freely accessed at http://sdd.whu.edu.cn/dpiki. It is running upon Linux/Apache/Dijango platform and supported by background Python language, which enables multiple accesses simultaneously. To evaluate the drug-target interaction, the users only need to input a drug molecule in the SMILES format and a target protein in the FASTA format, respectively. For convenience, the user is allowed to draw a drug molecule via JME editor. Examples with standard input formats are also provided to guide the users. After submission, the back-end server can calculate a 1191 dimensional vector representing the interaction, and then RF can give a predictive probability for this drug-target pair.

## Discussion

In modern genomic drug discovery, chemogenomics is urgently needed to screen potential drug candidates for clinical trials and to identify targets that have expected binding affinities [51], [52], [53]. In this study, we have shown that a RF can accurately predict the drug-target interactions based on integrated features, following the spirit of the chemogenomics approach. The application to several external validation sets has further demonstrated the reliability of our approach. When targets lack 3-D structures, our approach provides an effective and alternative way to study the action of drugs and targets.

When scanning the whole cross-linking set to predict the putative associations, we predict that 99% of 9659 drug-target interactions with RF probabilities above 0.95 are true positives. This is a conservative estimate of our ability to identify novel drug-target interactions. These predicted associations are useful for experimentalist, especially in solving problems related to drug-target selectivity and polypharmacology. Our network analysis demonstrates this point. In addition, the reliability of our chemogenomics framework is further demonstrated by only considering chemical space or genomics space. Clearly, combining two spaces could significantly improve the prediction of drug-target interactions, implying the close cooperation. Furthermore, analysis and comparison of protein features indicate the complexity of drug-target interactions and the difficulty of interpreting specific protein feature.

The main advantages of our proposed approach are summarized as follows: 1) The model directly encodes the drug-target pairs using integrated features called the pharmaceutical space. Application to RF effectively explores the complex interaction relationship in the pharmaceutical space. The system is suitable for simultaneously screening huge numbers of drug candidates and candidate targets from a systematic level; 2) Most previous algorithms are assumed that unknown interactions are considered as non-interactions (i.e., negative set); thereby cause a model bias which enables a large number of true interactions without experimental confirmation to be predicted as non-interactions (i.e., false negatives). However, a series of problems caused by such an assumption could be overcome by K_i_ values. 3) Compared with the structure-based simulation methods, this approach is not limited by the 3D structure data of targets, and it is also fast and convenient; 4) The approach can assist in discovery of multi-target drugs by recognizing the group of proteins targeted by a particular ligand. 5) Applying binding affinity data to prediction helps to distinguish strong drug-target interactions from those weak interactions or non-interactions, accelerating the discovery of drugs at μM even nM level. However, a limitation of our approach is that it may be insufficient or invalid to identify those interactions deviating from structural similarity principle since our approach is based on the assumption that the drug-target pairs with similar structure should have similar properties [54], [55]. Detecting such interactions may need the development of specific modeling approaches [12], [56], [57], [58].

(Quantitative) structure-activity relationship (QSAR/SAR), as a classical drug research approach, assumes that molecules with similar structure should exhibit similar activity. These classical QSAR/SAR models only take into account multiple molecules active in a single protein target, yet they completely neglect our extensive knowledge on the similarities of targets to each other and drug-target interactions. Therefore, they are not able to rationalize why an inhibitor is active on one protein but not on another [59], [60]. In fact, our chemogenomics framework can easily be considered as a natural extension of classical QSAR/SAR. At a reductionist level, our chemogenomics framework can be rationalized by similarities of key molecular fingerprints in drugs when the targets that bind drugs are invariant; and when comparing proteins from different families, the framework can be rationalized by similarities or differences in the physicochemistry properties of the residues of proteins (the drugs are invariant).

Our prediction approach offers several other applications. Predicted drug-target associations can guide experimental identification of drug-target interactions and may be used to infer protein function by predicting if a protein has similar function to its interacting partner(s). Application to binding-affinity-related endpoints for all drug-target pairs enables us to construct a more reliable and robust model. Identification of tight binding associations could also greatly accelerate the finding of drug molecules at μM even nM level. However, it should be noted that the ability of a protein to bind a small molecule with the appropriate chemical properties at the required binding affinity might make it druggable, but does not necessarily make it a potential drug target for that honor belongs only to proteins that are also linked to disease. This framework may also be useful in predicting drug-target interactions experimented on other species.

Certainly, the RF models developed in the current work are far from perfect, because the dataset used here is limited. A sufficiently accurate set of experimental data relevant to K_i_ for the validation is crucial in the development of the prediction models. So, based on increasing data, the learning/modeling will need to be an ongoing, iterative process in which the models are continuously refined.

## Materials and Methods

### Data sets of drug-target interactions

The training set was composed of 514 target proteins and 3393 drug-like ligands, with 13079 associated drug-target interactions. The drug-target interactions were extracted from the Binding database and the PDSP Ki database [61]. For each drug-target pair, we also extracted its corresponding K_i_ values in these two databases. Maybe these two databases included one or even several K_i_ values for one drug-target pair due to the integration of different sources. Thus, we used a median of these K_i_ values as a reference K_i_ to assure reliability. We defined a target as a protein that physically binds to the drug and a ligand as a compound that physically binds to the target protein. Although some target proteins in two databases also bind drug-like ligands, they were excluded from our training set because our main focus was to predict specific drug-target pairs in human species. We used a heuristic approach to identify only human-specific drug-target pairs by excluding those from other species, such as “rat”, “rabbit”, “bovine”, “sheep”, “calf”, “pig”, “mouse”, “guinea pig”, “dog” and “undefined”. These associations together with those drugs and targets are included in **Supporting Materials A–C in File S1.**

We also collected six independent validation sets from different sources to validate our model from different aspects. The first one is 2041 drug-target associations involving 435 effect-mediating targets and 989 drugs, which are extracted from the DrugBank database [62], [63]. Many of these have been approved and applied to the treatment of diseases. The second is a gold standard dataset released by Yamanishi et al. [64], which covers 5127 drug-target interactions involving 989 targets and 932 drugs. The third is 30102 drug-target interactions involving 295 targets and 12984 drugs from the ChEMBL database [44]. The fourth is 334 protein-ligand complexes involving 233 targets and 198 drugs from the AffinDB database [65]. The fifth is 1560 decoy protein-ligand complexes involving 39 targets and 1545 drugs from the DUD database [66]. The final is from the research work of Keiser and Campillos [13], [67]. 43 drug-target associations were masterly discovered by two different screening strategies and were then further confirmed by *in vitro* binding assays. All drugs and target proteins used in the validation are included in **Supporting Materials D–I in File S1.** The number of drugs, targets and their interactions in the training set and independent validation sets are listed in **Table S1.**

### Random forest

RF, developed by Bremain and Culter [68], is capable of describing the relationship between independent and dependent variables with high flexibility and sufficient accuracy. An extended depiction and study of theory on RF can be referred to the Web site of Bremain or the papers of Svetnik et al. [69]. The RF algorithm grows a collection, called a forest, of the unpruned classification trees and uses these for classifying a data point into one of the classes. Two types of randomness, bootstrap sampling of samples and random selection of input features, are used in the algorithm to make sure that the classification trees grown in the forest are dissimilar and uncorrelated from each other. A forest is grown by using *ntree* bootstrapped samples, each of size *N* randomly drawn from the original data of *N* training samples with replacement. This first type of randomization helps to build an ensemble of trees and to increase diversity among the trees. In each bootstrap sample, about two-thirds of the original training samples are used to grow a classification tree. About one-third of the samples are left, called Out Of Bag (OOB) samples. These samples are used to obtain unbiased estimates of correct classification rates and feature importance measure. The second type of randomness is used during building each tree. For each node of a tree, the RF algorithm randomly selects *mtry* features and uses only them to determine the best possible split using the *Gini* index as the splitting criterion [70]. Predictions for test data are carried out either by the majority vote of classification trees or are based on a threshold selected by the user. The number of trees (*ntree*) to be grown is chosen appropriately to achieve low error rate of convergence. Furthermore, RF includes a method for assessing the importance of features in the model. When each feature is replaced in turn by random noise, then the resulting deterioration in model quality is a measure of variable importance. The deterioration in model quality can be assessed by the change in misclassification rates for the OOB validation. Finally, RF can produce scores or probability outputs that serve to rank predictions according to confidence and have a useful probabilistic interpretation.

### Representing drug molecules and protein targets

For drug descriptors, the open-source OpenBabel was used to calculate two-dimensional topological Daylight fingerprints using default settings of 1024 bits array length and path lengths of 2–7 atoms. Proteins are represented using amino acid composition descriptors and CTD descriptors (composition, transition and distribution) [71]. Amino acid composition descriptors reflect the fraction of each amino acid type in a protein sequence. Composition is the number of amino acids of a particular property (e.g., hydrophobicity) divided by the total number of amino acids in a protein sequence. Transition characterizes the percent frequency with which amino acids of a particular property is followed by amino acids of a different property. Distribution measures the chain length within which the first, 25%, 50%, 75%, and 100% of the amino acids of a particular property are located, respectively.

The CTD descriptors can be calculated as follows: Firstly, the sequence of amino acids for a protein sample is transformed into sequences of certain structural or physiochemical properties of residues. In this work, seven feature properties are used to describe the physiochemical characteristics of each amino acid, which have been used routinely for the prediction of protein-related problems. The ranges of these numerical values and the amino acids belonging to each group are shown in **Table S2**. Twenty amino acids are thus divided into three groups representing the main clusters of the amino acid indices [72]. For each attribute, every amino acid is replaced by the index ‘1’, ‘2’, or ‘3’ according to one of three groups to which it belongs. Take MTEITAAMVKELRESTGAGA for an example; according to hydrophobicity, its amino acid sequence is encoded as: 32132223311311222222. A schematic diagram indicating the construction process of three descriptors is shown in **Figure S7**. There are five ‘1’, ten ‘2’ and five ‘3’ in this protein sequence. The composition for three symbols is n1×100.00/(n1+n2+n3)  = 25.00, n2×100.00/(n1+n2+n3)  = 50.00 and n3×100.00/(n1+n2+n3)  = 25.00, respectively. There are 2 transitions from ‘1’ to ‘2’ or from ‘2’ to ‘1’ in this sequence, and the percent frequency of these transitions is (2/19)×100.00 = 10.53. The transitions from ‘1’ to ‘3’ or from ‘3’ to ‘1’ in this sequence can similarly be calculated as (4/19) ×100.00 = 21.05. The transitions from ‘2’ to ‘3’ or from ‘3’ to ‘2’ in this sequence can also similarly be calculated as (3/19) ×100.00 = 15.79. For distribution D, for example, there are 10 residues encoded as “2”, the positions for the first residue ‘2’, the 2th residue ‘2’ (25%×10 = 2), the 5th ‘2’ residue (50%×10 = 5), the 7th ‘2’ (75%×10 = 7) and the 10th residue ‘2’ (100%×10) in the encoded sequence are 2, 5, 15, 17,20 respectively, so the D descriptors for ‘2’ are: 10.0 (2/20×100), 25.0 (5/20×100), 75.0 (15/20×100), 85.0 (17/20×100), 100.0 (20/20×100), respectively. Likewise, the D descriptor for ‘1’ and ‘3’ is (15.0, 15.0, 50.0, 55.0, 70.0) and (5.0, 5.0, 20.0, 40.0, 60.0), respectively. Overall, the CTD descriptors for this sequence are C =  (25.0, 50.0, 25.0), T =  (10.53, 21.05, 15.79), and D =  (15.0, 15.0, 50.0, 55.0, 70.0, 10.0, 25.0, 75.0, 85.0, 100.0, 5.0, 5.0, 20.0, 40.0, 60.0). Thus, for each given structural or physiochemical property of residues, we can obtain 3+3+15 = 21 protein descriptors. Descriptors for other properties can be computed by a similar procedure, and a total of 147 descriptors are calculated to form the feature vector. Finally, a total set of 167 protein descriptors were obtained.

## Supporting Information

File S1 **The reference set used for constructing RF models and the validation sets used for validating RF models.** A: the reference set; B: drugs in the reference set; C: targets in the reference set; D: validation set 1 from DrugBank; E: validation set 2 from KEGG; F: validation set 3 from ChEMBL; G: validation set 4 from AffinDB; H: validation set 5; I: validation 6; J: 11907 predicted drug-target interactions at a RF threshold of 0.95; K: 775 new drug-target interactions at a RF threshold of 0.99.
(XLS)

Figure S1 **The plot of OOB error rate versus two tuned parameters in the RF model:** ***ntree*** **and** ***mtry*** **, respectively.** (A) 1200 classification trees are grown to seek for a suitable *ntree* value. About RF model of 600 trees can achieve a low OOB error rate of convergence. (B) *mtry* values in the range from 5 to 100 with a step of 5 are screened to find a low OOB error rate. For each *mtry* value, we run the RF model five times to obtain a stable OOB error rate. We finally select *mtry* = 90 to construct our RF model.
(TIF)

Figure S2 **Probability density of prediction probability for different K_i_ thresholds.**
(TIF)

Figure S3 **Performance comparison when different protein properties are omitted.** Number 1 corresponds to the auROC value for the full feature set. Number 2–8 corresponds to the auROC value when hydrophobicity, normalized van der Waals volume, polarity, polarizability, charge, secondary structure, solvent accessibility and amino acid composition are omitted, respectively.
(TIF)

Figure S4 **Receiver operator characteristics curve on 5-fold cross validation data using integrated features, drug features and protein features, respectively.**
(TIF)

Figure S5 **Drug-target interaction network using both predicted drug-target pairs and those in the training set.** Drugs and targets are presented by red circle and blue triangle, respectively. Drug-target interactions are represented by the edges connecting related drugs and targets.
(TIF)

Figure S6 **Receiver operator characteristics curve on 5-fold cross validation data using four modeling algorithms.** For SVM, the parameters gamma and cost are tuned over an exponential range. For BPN, principal component analysis (PCA) is first used for extracting the first few principal components (PCs) that explain variations of 95%, and then standard three-layer BPN algorithm is performed in these PCs as input. The number of hidden nodes is scanned from 2 to 10. For *k*-NN, the size of *k* is scanned from 1 to 9 with step of 2.
(TIF)

Figure S7 **Sequence of a hypothetic protein indicating the construction of composition, transition and distribution descriptors of a protein.** Sequence index indicates the position of an amino acid in the sequence. The index for each type of amino acids in the sequence (‘1’ ‘2’ or ‘3’) indicates the position of the first, second, third, ... of that type of amino acid. 1/2 transition indicates the position of ‘12’ or ‘21’ pairs in the sequence (1/3 and 2/3 are defined in the same way).
(TIF)

Table S1 **The number of drugs, targets and interactions in the training set and independent validation sets.**
(DOC)

Table S2 **Amino acid attributes and the division of the amino acids into three groups for each attribute.**
(DOC)
