# Supplementary figures and images for: Genome-Scale Screening of Drug-Target Associations Relevant to Ki Using a Chemogenomics Approach
Source: PLoS One. 2013 Apr 5;8(4):e57680. doi: 10.1371/journal.pone.0057680 (PMC3618265; doi:10.1371/journal.pone.0057680)

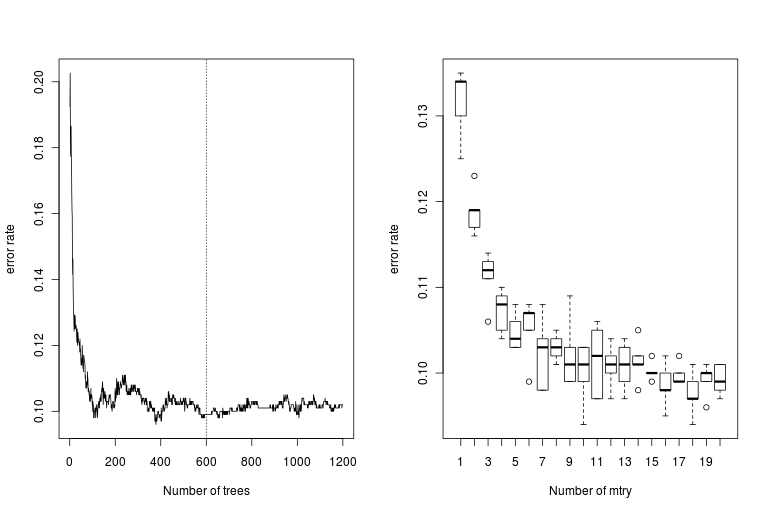

Supplement: Figure S1 — The plot of OOB error rate versus two tuned parameters in the RF model: ntree and mtry , respectively. (A) 1200 classification trees are grown to seek for a suitable ntree value. About RF model of 600 trees can achieve a low OOB error rate of convergence. (B) mtry values in the range from 5 to 100 with a step of 5 are screened to find a low OOB error rate. For each mtry value, we run the RF model five times to obtain a stable OOB error rate. We finally select mtry = 90 to construct our RF model. (TIF) [file pone.0057680.s002.tif]

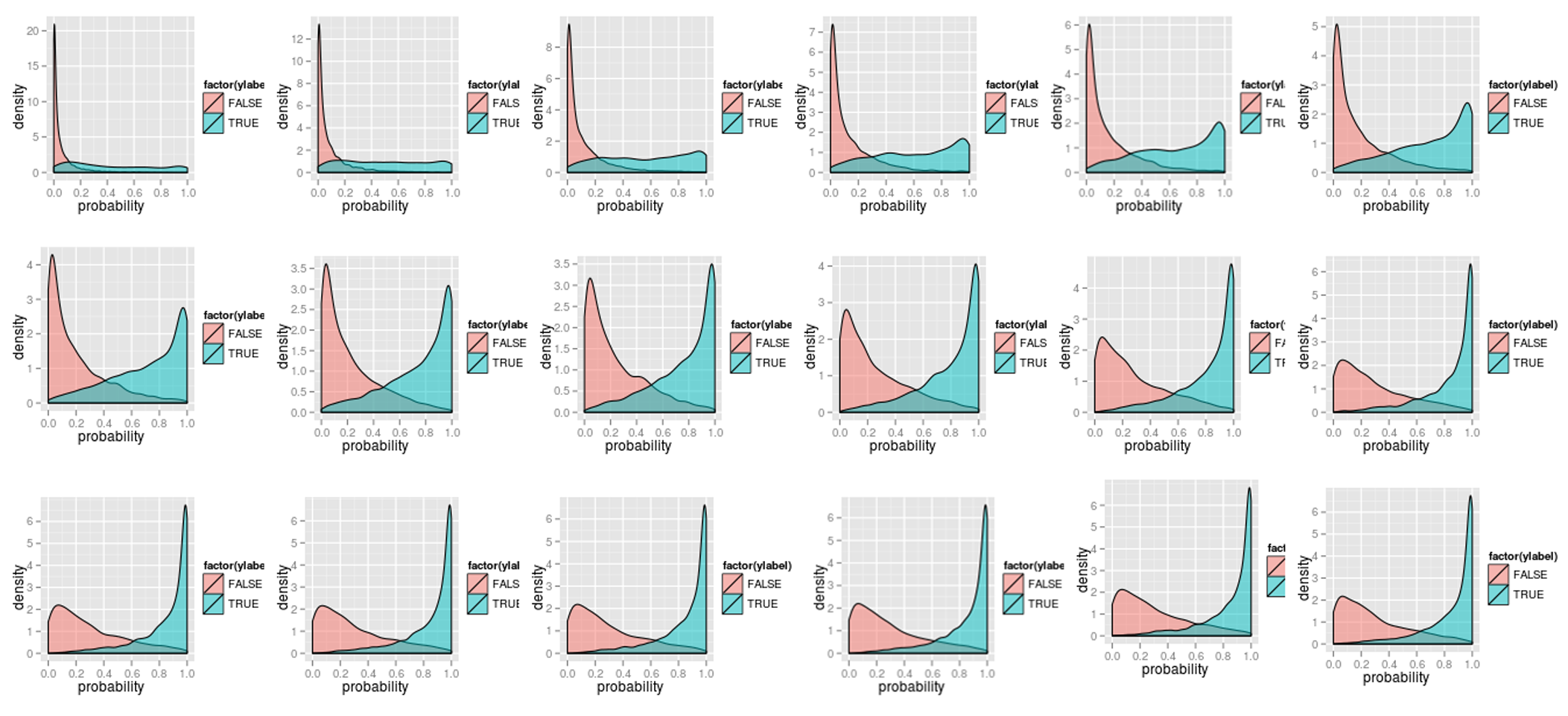

Supplement: Figure S2 — Probability density of prediction probability for different Ki thresholds. (TIF) [file pone.0057680.s003.tif]

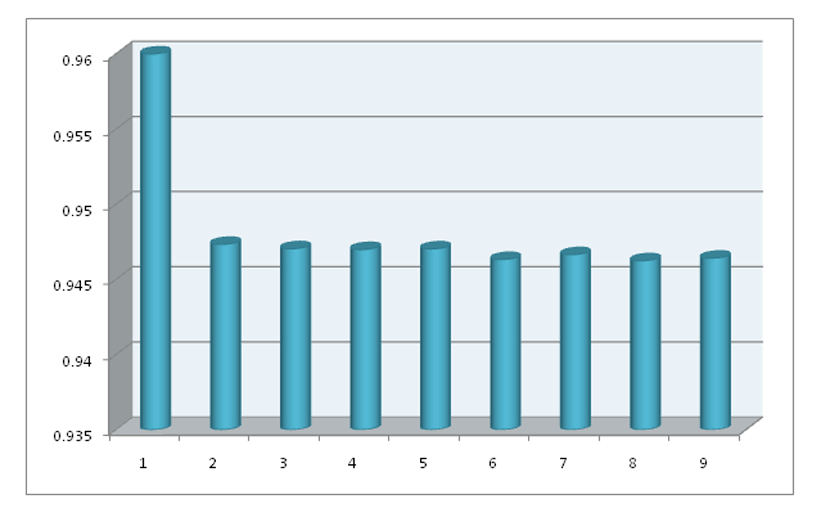

Supplement: Figure S3 — Performance comparison when different protein properties are omitted. Number 1 corresponds to the auROC value for the full feature set. Number 2–8 corresponds to the auROC value when hydrophobicity, normalized van der Waals volume, polarity, polarizability, charge, secondary structure, solvent accessibility and amino acid composition are omitted, respectively. (TIF) [file pone.0057680.s004.tif]

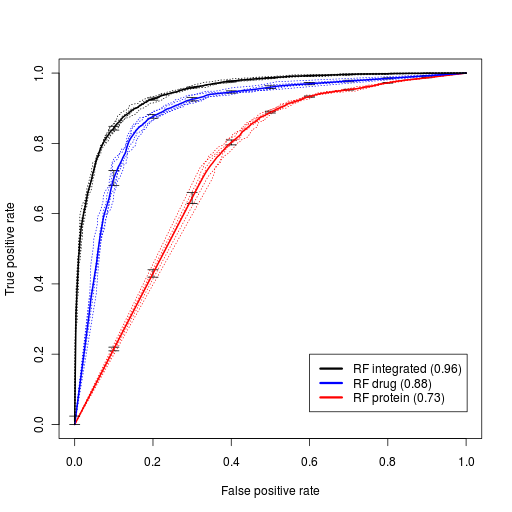

Supplement: Figure S4 — Receiver operator characteristics curve on 5-fold cross validation data using integrated features, drug features and protein features, respectively. (TIF) [file pone.0057680.s005.tif]

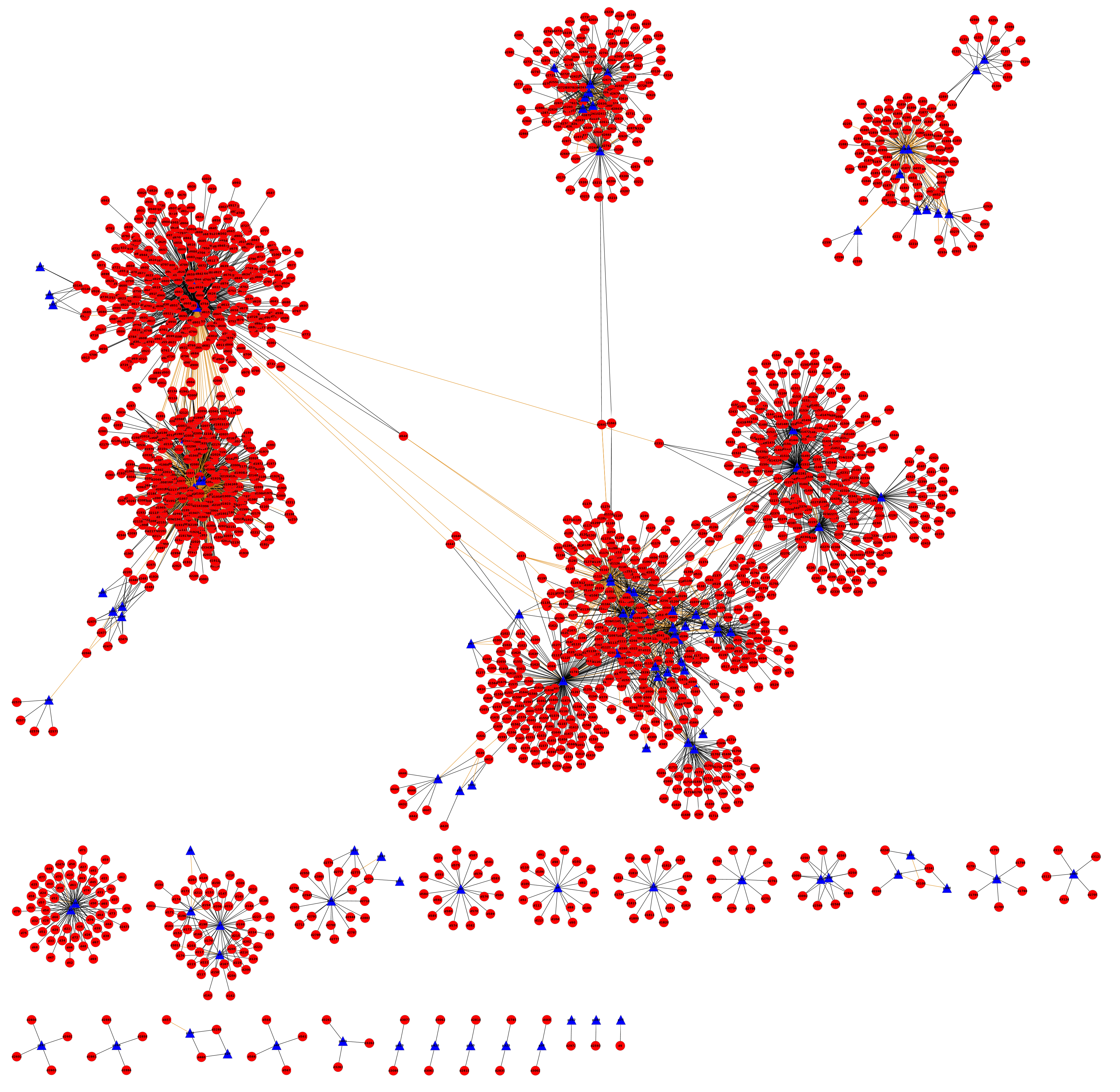

Supplement: Figure S5 — Drug-target interaction network using both predicted drug-target pairs and those in the training set. Drugs and targets are presented by red circle and blue triangle, respectively. Drug-target interactions are represented by the edges connecting related drugs and targets. (TIF) [file pone.0057680.s006.tif]

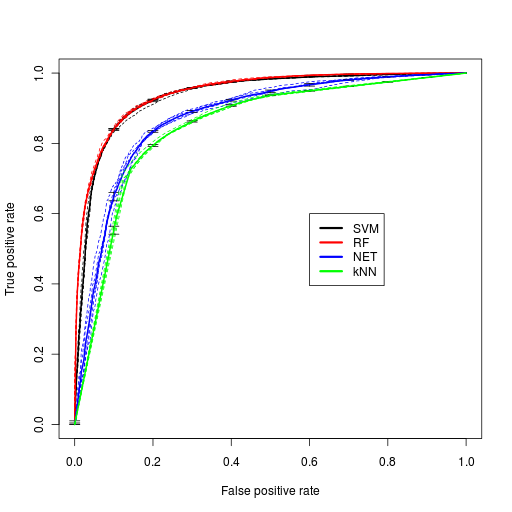

Supplement: Figure S6 — Receiver operator characteristics curve on 5-fold cross validation data using four modeling algorithms. For SVM, the parameters gamma and cost are tuned over an exponential range. For BPN, principal component analysis (PCA) is first used for extracting the first few principal components (PCs) that explain variations of 95%, and then standard three-layer BPN algorithm is performed in these PCs as input. The number of hidden nodes is scanned from 2 to 10. For k-NN, the size of k is scanned from 1 to 9 with step of 2. (TIF) [file pone.0057680.s007.tif]

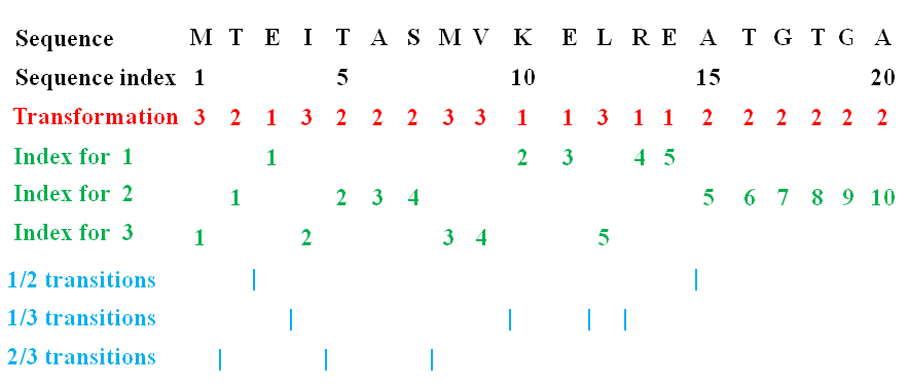

Supplement: Figure S7 — Sequence of a hypothetic protein indicating the construction of composition, transition and distribution descriptors of a protein. Sequence index indicates the position of an amino acid in the sequence. The index for each type of amino acids in the sequence (‘1’ ‘2’ or ‘3’) indicates the position of the first, second, third, ... of that type of amino acid. 1/2 transition indicates the position of ‘12’ or ‘21’ pairs in the sequence (1/3 and 2/3 are defined in the same way). (TIF) [file pone.0057680.s008.tif]
