# Supplementary material for: Genome-Scale Screening of Drug-Target Associations Relevant to Ki Using a Chemogenomics Approach
Source: PLoS One. 2013 Apr 5;8(4):e57680. doi: 10.1371/journal.pone.0057680 (PMC3618265; doi:10.1371/journal.pone.0057680)
Supplement: Table S1 — The number of drugs, targets and interactions in the training set and independent validation sets. (DOC) [file pone.0057680.s009.doc]

**Table S1** The number of drugs, targets and interactions in the training set and independent validation sets

|  | Number of drugs | Number of targets | Number of interactions |
| --- | --- | --- | --- |
| Training set | 3393 | 514 | 13079 |
| Validation set 1 | 989 | 435 | 2041 |
| Validation set 2 | 932 | 989 | 5127 |
| Validation set 3 | 12984 | 295 | 30102 |
| Validation set 4 | 198 | 233 | 334 |
| Validation set 5 | 1545 | 39 | 1560 |
| Validation set 6 | 26 | 23 | 43 |
