# Supplementary material for: Genome-Scale Screening of Drug-Target Associations Relevant to Ki Using a Chemogenomics Approach
Source: PLoS One. 2013 Apr 5;8(4):e57680. doi: 10.1371/journal.pone.0057680 (PMC3618265; doi:10.1371/journal.pone.0057680)
Supplement: Table S2 — Amino acid attributes and the division of the amino acids into three groups for each attribute. (DOC) [file pone.0057680.s010.doc]

**Table S2** Amino acid attributes and the division of the amino acids into three groups for each attribute

|  | **Group 1** | **Group 2** | **Group 3** |
| --- | --- | --- | --- |
| hydrophobicity | Polar  R,K,E,D,Q,N | Neutral  G, A, S,T,P,H,Y | Hydrophobicity  C,L,V,I,M,F,W |
| normalized van der Waals volume | 0-2.78  G,A,S,T,P,D | 2.95-4.0  N,V,E,Q,I,L | 4.03-8.08  M,H,K,F,R,Y,W |
| polarity | 4.9-6.2  L,I,F,W,C,M,V,Y | 8.0-9.2  P,A,T,G,S | 10.4-13.0  H,Q,R,K,N,E,D |
| polarizability | 0-1.08  G,A,S,D,T | 0.128-0.186  C,P,N,V,E,Q,I,L | 0.219-0.409  K,M,H,F,R,Y,W |
| charge | Positive  K,R | Neutral  A,N,C,Q,G,H,I,L,M,F,P,S,T,W,Y,V | Negative  D,E |
| secondary structure | Helix  E,A,L,M,Q,K,R,H | Strand  V,I,Y,C,W,F,T | Coil  G,N,P,S,D |
| solvent accessibility | Buried  A,L,F,C,G,I,V,W | Exposed  R,K,Q,E,N,D | Intermediate  M,S P,T,H,Y |
